# Novel Insights into Beta 2 Adrenergic Receptor Function in the rd10 Model of Retinitis Pigmentosa

**DOI:** 10.3390/cells9092060

**Published:** 2020-09-09

**Authors:** Maurizio Cammalleri, Massimo Dal Monte, Rosario Amato, Dominga Lapi, Paola Bagnoli

**Affiliations:** Department of Biology, University of Pisa, 56127 Pisa, Italy; maurizio.cammalleri@unipi.it (M.C.); rosario.amato@biologia.unipi.it (R.A.); dominga.lapi@unipi.it (D.L.)

**Keywords:** retina degenerative disease, sympathetic transmission, desensitization, HIF-1α stabilization, inflammatory process, cone loss, retinal function

## Abstract

**Background**: In retinitis pigmentosa (RP), inherited rod death is followed by cone loss and blindness. Why cones die is still a matter of consideration. Here, we investigate the pathogenic role of the sympathetic transmission in the rd10 mouse model of RP. **Methods**: Retinal levels of beta adrenergic receptor (BAR) 2 and norepinephrine (NE) were measured. After administration of the BAR1/2 blocker propranolol or the hypoxia-inducible factor (HIF)-1 activator dimethyloxalylglycine (DMOG), retinal levels of HIF-1α, BAR2 or proteins involved in BAR2 desensitization were also measured. In DMOG treated mice, expression and localization of BAR2, inflammatory markers and cone arrestin were determined. Finally, rd10 mice were subjected to electroretinogram (ERG) analysis to assess rod and cone function. **Results**: In the rd10 retina, BAR2 overexpression and NE accumulation were found, with BAR2 immunoreactivity localized to Müller cells. BAR2 overexpression was likely due to desensitization defects. Upregulated levels of BAR2 were drastically reduced by propranolol that also restored desensitization defects. Due to the low level of HIF-1 consequent to the hyperoxic environment in the rd10 retina, we hypothesized a link between HIF-1 and BAR2. HIF-1α stabilization with DMOG resulted in i. increased HIF-1α accumulation, ii. decreased BAR2 levels, iii. restored desensitization processes, iv. reduced expression of inflammatory markers and v. increased cone survival without improved retinal function. **Conclusions**: Our results support a pathogenic role of the sympathetic system in RP that might help to understand why rd10 mice show a positive response to BAR blockers.

## 1. Introduction

Retinitis pigmentosa (RP) refers to a family of genetically determined retinal diseases characterized by progressive and irreversible loss of vision due to rod inherited death followed by cone degeneration [1]. Why cones die following rods in RP is still a matter of consideration. Evidence suggests that inappropriate oxygen levels may contribute to the pathogenesis of this disease [2]. Indeed, rod death results in decreased oxygen consumption that leads to a large excess of oxygen in the outer retina. For instance, in a rat model of RP, the oxygen tension in the outer retina is about 50% higher than in wild type (WT) rats [3]. This hyperoxic state is accompanied by reduced levels of the transcription factor hypoxia-inducible factor (HIF)-1, which responds to oxygen levels in the retinal environment [4]. In particular, mouse models of RP are characterized by HIF-1 levels that are about 50% lower than those measured in WT mice [5,6]. In experimental RP, early stabilization of the labile α subunit of HIF-1 (HIF-1α) with dimethyloxalylglycine (DMOG), an inhibitor of prolyl hydroxylases that avoids the hydroxylation of HIF-1α and its consequent degradation [7], prevents photoreceptor cell death, neuroinflammation and oxidant responses, suggesting a link between HIF-1 levels and retinal degeneration [5,6,8].

Recently, attention has been paid to the study of the role of the noradrenergic system in ocular diseases in which stress plays an important role through the release of catecholamines acting at beta adrenergic receptors (BARs). Preclinical evidence demonstrates that both rd10 mice and oxygen induced retinopathy (OIR) mice, used as models of RP and retinopathy of prematurity, respectively [9,10], show a positive response to BAR blockers acting mainly at BAR1 and BAR2 [11,12,13,14]. In particular, in the rd10 model, the BAR1/2 blocker metipranolol efficiently counteracts cone degeneration by reducing nitrosative damage to cones [11], while, in the OIR model, the BAR1/2 blocker propranolol prevents retinal neovascularization and dysfunction by reducing angiogenic processes [12,13,14]. Metipranolol and propranolol display some preferential selectivity for BAR2 [15,16]. Both may be used in the treatment of arterial hypertension given their efficacy to block BAR2 expressed by the vascular smooth muscle cells where BAR2 is activated by norepinephrine (NE) released by sympathetic nerves or circulating epinephrine.

In the retina, NE is mainly released by the sympathetic nerves although it may also be produced endogenously by amacrine and endothelial cells [17]. NE activates BAR2, which is localized to Müller cells as identified by glutamine synthetase immunoreactivity [18] and to other cell types roughly postulated to as bipolar and amacrine cells given their localization to the inner retina [17]. BAR2 localization to Müller cells is in line with previous results demonstrating that cultured Müller cells of the rat retina express BAR2 immunoreactivity [19].

Although previous evidence indicates that sympathetic transmission significantly contributes to retinal diseases [17], the mechanisms underlying the potential efficacy of BAR blockers remain to be clarified. One possibility is that BAR2 levels are coupled to retinal damage through the intervention of HIF-1. Our hypothesis is that HIF-1 regulates the expression of BAR2 by acting on ligand-induced receptor desensitization. After being activated by its agonists, BAR2 undergoes phosphorylation by several kinases of which G protein-coupled receptor kinase (GRK)2 is the prototype GRK for BAR2 desensitization [20]. Following its phosphorylation, BAR2 becomes able to interact with members of the arrestin family, in particular with β-arrestin 2, thus triggering internalization and desensitization [21]. Our additional hypothesis is that dysregulated BAR2 may participate in the neuroinflammatory process leading to cone loss in RP, as an important correlation between the BAR system activity and inflammatory processes has been demonstrated in the diseased retina [22].

The present study was aimed at understanding the pathogenic role of the sympathetic transmission in the diseased retina using the rd10 mouse as a model of photoreceptor degeneration conjugated to hyperoxic retinal environment. Transcript and protein levels of BAR2 were determined together with retinal levels of NE, and mechanisms regulating BAR2 expression were also investigated. In particular, the developmental expression of BAR2 was studied in concomitance with that of the main desensitization markers GRK2 and β-arrestin 2 in order to correlate BAR2 levels and their regulatory mechanisms with major events leading to cone death. Although the efficacy of metipranolol on nitrosative damage to cones has been previously reported [11], no information is available on whether BAR blockade affects the BAR system. Here, the effects of BAR blockade on BAR2, GRK2 and β-arrestin 2 are also determined in order to evaluate whether blocking BAR2 might affect desensitization processes. The possibility that HIF-1 regulates the expression of BAR2 by acting on receptor desensitization was tested by investigating whether recovering HIF-1 levels by DMOG administration to prevent HIF-1α degradation might affect the levels of BAR2, GRK2 and β-arrestin 2. Whether sympathetic overactivation is correlated with the pathogenic state of the retina was also determined by evaluating if preventing dysregulated levels of BAR2 and its desensitization markers might restore the inflammatory responses triggered by rod inherited death and to rescue cone photoreceptors from loss.

## 2. Materials and Methods

### 2.1. Experimental Animals

C57BL/6J mice (used as WT controls) and rd10 mutants (B6.CXB1-Pde6brd10/J on a C57Bl6J background) [23] were obtained from Charles River Laboratories, Italia (Calco, Italy) and mated in our breeding colonies. Experimental animals were housed in a regulated environment (23 ± 1 °C, 50 ± 5% humidity) with a 12 h light/dark schedule (lights on at 08:00 a.m.) and provided with a standard diet and water ad libitum. In rd10 mice, the presence of the homozygous Pde6b mutation was assessed periodically with PCR on DNA extracted from tail tissue. Overall, 110 mice (45 WT, 22 males and 23 females, and 65 rd10, 36 males and 29 females) were used. Of the WT mice, 35 were left untreated (5 sacrificed at postnatal day (PD) 9, 5 at PD15 and 25 at PD30), 5 were propranolol-treated and 5 received the vehicle in which propranolol was dissolved. Of the rd10 mice, 35 were left untreated (5 sacrificed at PD9, 5 at PD15 and 25 at PD30), 5 were treated with propranolol, 5 received the vehicle in which propranolol was dissolved, 10 were treated with DMOG and 10 received the vehicle in which DMOG was dissolved. In all experiments, mice were anesthetized using isoflurane before sacrifice and then humanely euthanized by cervical dislocation. To evaluate retinal function by electroretinography, 5 mice at PD30 were randomly chosen in the groups of untreated WT, untreated rd10 and rd10 treated with DMOG or its vehicle. Animal studies were carried out in compliance with the recommendations in the Guide for the Care and Use of Laboratory Animals of the National Institutes of Health, the Association for Research in Vision and Ophthalmology Statement for the Use of Animals in Ophthalmic and Vision Research, the Italian guidelines for animal care (DL 6/14), and the European Communities Council Directive (2010/63/EU). The experimental procedures were approved by the Ethical Committee in Animal Experiments of the University of Pisa (permission number: 0034612/2017). All efforts were made to reduce animal suffering and the number of animals required to obtain reliable results based on the rule of the replacement, refinement and reduction (the 3Rs).

### 2.2. Treatments

Treatments with propranolol or DMOG were initiated at PD14 and continued daily until PD30. Propranolol (Sigma-Aldrich, St. Louis, MO, USA) was dissolved in citrate buffer. It was given subcutaneously 3 times a day at 20 mg/kg/dose, based on previous findings demonstrating that this regimen of systemic propranolol was beneficial on the diseased retina without any effect in the brain or those organs, such as lungs and heart, known to be targeted by BAR blockers [12,14]. DMOG (Cayman Chemical, East Ellsworth, MI, USA) was dissolved in dimethyl sulfoxide, diluted in phosphate-buffered saline (PBS) at 1:100 and introperitoneally injected at 200 mg/kg in line with previous studies in the mouse retina [6].

### 2.3. Quantitative Real Time PCR

For transcript measurements, eyes were enucleated, the retinas were separated from the eyecups and stored at −80 °C. Five samples, each containing 2 retinas from independent WT or rd10 mice, were used. Total RNA was extracted (TRI reagent, Sigma-Aldrich), according to the manufacturer instructions. First-strand cDNA was produced from 1 µg of total RNA (QuantiTect Reverse Transcription Kit, Qiagen, Valencia, CA, USA). The SsoAdvanced Universal SYBR Green Supermix (Bio-Rad Laboratories, Hercules, CA, USA) was used to perform real-time PCR amplifications on a CFX Connect Real-Time PCR detection system equipped with the software CFX manager (Bio-Rad Laboratories). Primer pairs were chosen to hybridize to unique regions of the appropriate gene sequence. The primer sequences are reported in Table S1. Amplification efficiency was close to 100 % for each primer pair. The PCR protocol was as follows: 1 cycle of denaturation at 95 °C for 10 min and 40 cycles of amplification (20 s of denaturation at 95 °C, 20 s of annealing at 58 °C and 30 s of extension at 72 °C). This last step was followed by a melting curve analysis from 40 to 95 °C and afterwards cooling to room temperature. Each sample was analyzed in triplicate. Target genes were assayed concurrently with *Rpl13a* used as endogenous control. The *Rpl13a* gene encodes a ribosomal protein that is a component of the 60S subunit [24] and has been validated as a stable housekeeping gene in the retina [18]. In addition, it has been demonstrated that, among several candidate housekeeping genes, *Rpl13a* is one of the most stable in hyperoxic conditions [25]. Transcripts were quantified using the ΔΔCt method.

### 2.4. Western Blotting

For protein measurements, eyes were enucleated, the retinas were separated from the eyecups and stored at −80 °C. Five samples per group, each containing 2 retinas from independent mice, were used. RIPA lysis buffer (50 mM Tris, pH 7.4 containing 150 mM NaCl, 1% Triton X-100, 1% sodium deoxycholate, 0.1% SDS, 5 mM EDTA) added with proteinase and phosphatase inhibitor cocktails (Roche Applied Science, Indianapolis, IN, USA) was used to extract proteins. The Micro BCA Protein Assay (Thermo Fisher Scientific, Waltham, MA, USA) was used to quantify protein content.

In separate experiments, to evaluate BAR2 protein levels in the cytosol and in the plasma membrane independently, 5 additional samples from WT or rd10 mice (each containing 2 retinas from independent mice) were lysed using the homogenization buffer, added with protease inhibitors, contained in a plasma membrane protein extraction kit (Abcam, Cambridge, UK). After homogenization, samples were centrifuged at 700× *g* for 10 min at 4 °C, collecting the supernatant that was then further centrifuged at 10,000× *g* for 30 min at 4 °C. The supernatant, containing the cytosolic fraction, was collected while the pellet was used to purify plasma membrane proteins according to the manufacturer’s protocol. The Micro BCA Protein Assay (Thermo Fisher Scientific) was used to quantify protein content.

Thirty micrograms of proteins for each sample were subjected to SDS-PAGE (4–20%; Bio-Rad Laboratories). Gels were transblotted onto a PVDF membrane (Bio-Rad Laboratories). Blots were blocked in 3% skim-milk for 1 h at room temperature and incubated overnight at 4 °C with antibodies listed in Table S2. Blots were then incubated for 1 h at room temperature with HRP-conjugated secondary antibodies (rabbit anti-mouse sc-2005, goat anti-rabbit sc-2004 or rabbit anti-goat sc-2768; all from Santa Cruz Biotechnology, Santa Cruz, CA, USA; all at 1:5000) and developed with Clarity Western enhanced chemiluminescence substrate (Bio-Rad Laboratories). Images were acquired (ChemiDoc XRS^+^; Bio-Rad Laboratories), and the optical density of the bands was evaluated (Image Lab 6.0 software; Bio-Rad Laboratories). The data were normalized to β-actin or Na^+^/K^+^ ATPase, as appropriate. All experiments were performed in duplicate.

### 2.5. Measurement of NE Levels

Five samples, each containing 2 retinas from independent WT or rd10 mice, were sonicated in a solution containing 0.1 M HCl and 1 mM EDTA. The homogenate was centrifuged at 22,000× *g* for 15 min at 4 °C. The Micro BCA Protein Assay (Thermo Fisher Scientific) was used to quantify protein content. NE levels were measured using a commercially available ELISA kit (IBL International, Hamburg, Germany). The ELISA plate was evaluated spectrophotometrically (Microplate Reader 680 XR; Bio-Rad Laboratories). Data were expressed as picomoles NE per milligram of proteins.

### 2.6. Immunohistochemistry and Quantitative Analysis

Eye-cups were immersion-fixed for 1.5 h in 4% paraformaldehyde in 0.1 M PBS at 4 °C, transferred to 25% sucrose in 0.1 M PBS and stored at 4 °C. Retinal sections (10 µm thick) were cut on a cryostat, mounted onto positive charged slides and stored at −20 °C until use. For immunostaining, mounted sections were rinsed in PBS and incubated overnight with the rabbit polyclonal anti-BAR2 (PA5-14117, Thermo Fisher Scientific; dilution 1:200), the rabbit monoclonal anti-glial fibrillary acid protein (GFAP; ab207165, Abcam; dilution 1:200), the rabbit monoclonal anti-ionized calcium-binding adapter molecule 1 (Iba1; ab178846, Abcam; dilution 1:500) and the rabbit polyclonal anti-cone arrestin (AB15282, Sigma-Aldrich; dilution 1:100) antibodies diluted in 0.1% *v*/*v* Triton X-100 in PBS. After being rinsed in PBS, the sections were incubated with appropriated secondary antibodies conjugated with Alexa-Fluor 488 (green; ab150077, Abcam; dilution 1:200) or Alexa-Fluor 555 (red; ab150078, Abcam; dilution 1:200) for 2 h at room temperature. The slides were coverslipped with Fluoroshield Mounting Medium containing 4′,6-diamidino-2-phenylindole (DAPI; Abcam) to visualize the nucleated layers of the retina. The images acquisition was performed using an epifluorescence microscope (Ni-E; Nikon-Europe, Amsterdam, The Netherlands) and immunofluorescent images were acquired using a digital camera (DS-Fi1c; Nikon-Europe). A scanning of whole retinal sections was carried out with a 10× plan apochromat objective. Images were then turned into grayscale, normalized for the background and analyzed for the mean gray levels to quantify the immunofluorescence intensity for each marker in 4 sections per experimental group using the analysis tool of Adobe Photoshop. The quantification of the outer nuclear layer (ONL) thickness was performed by sampling 4 areas in each section. Representative images of each immunostaining were acquired using a 20× plan apochromat objective and merged with the corresponding DAPI.

### 2.7. Measurement of Scotopic and Photopic Electroretinogram

Retinal function was examined at PD30 with scotopic and photopic full-field electroretinogram (ERG). Mice were dark adapted overnight and anesthetized by intraperitoneal injection of avertin (1.2% tribromoethanol and 2.4% amylene hydrate in distilled water, 0.02 mL/g body weight; Sigma-Aldrich). Pupils were dilated with a topical drop of 0.5% atropine. A heating pad was used to keep the body temperature at 38 °C. The electrophysiological signals were recorded through silver/silver chloride ring electrodes inserted under the lower eyelids. The cornea was intermittently irrigated with saline solution to prevent clouding of the ocular media. Electrodes in each eye were referred to a needle electrode inserted subcutaneously at the level of the corresponding frontal region. The ground electrode was inserted subcutaneously in the tail. The electrodes were connected to a two-channel amplifier. The light stimulation device consisted in Ganzfeld stimulator (Biomedica Mangoni, Pisa, Italy), which ensures a homogeneous illumination anywhere in the retina. Responses were collected simultaneously from both eyes, amplified at 1000 gain and filtered with a bandpass of 0.2 to 500 Hz before being digitized at 5 kHz rate with a data acquisition device (Biomedica Mangoni). Initially, the electrical recordings were taken without any stimulus in order to measure the background noise levels. The scotopic responses, which primarily reflect rod function although in the presence of a not negligible cone-driven contribution, were evoked by a 1.00 log cd-s/m^2^ flash intensity. After the completion of scotopic stimulation, photopic, cone-mediated responses, were recorded following 10-min light adaptation on the background light intensity of 30 cd/m^2^. Recordings were obtained at the light intensity of 10 cd-s/m^2^. From each animal, 10 waveforms were recorded and the values were averaged. The interval between light flashes was adjusted to appropriate times that allowed response recovering (20 s for scotopic responses, 3 s for photopic responses). All ERG waveforms were analyzed using a customized program (Biomedica Mangoni). The signals were filtered using a butterworth second order bandpass filter from 1 to 300 Hz and the signals averaged. In compliance with the International Society for Clinical Electrophysiology guidelines, the b-wave amplitude was measured from the trough of the a-wave to the peak of the b-wave or, if no a-wave was present, from the prestimulus baseline. For the noise measurement, root mean square of noise amplitude was measured.

### 2.8. Data Analysis

Experimental analysis and data collection were performed in a blinded fashion whenever possible. Blinding was performed by assigning a numerical coded identifier to the samples. Unblinding was only done following analysis. The sample size was chosen based on previous studies using the rd10 model analyzed here in order to ensure adequate statistical power. For the statistical analysis, Graph Pad Prism 8.0.2 was used (GraphPad Software, Inc., San Diego, CA, USA). All data are expressed as means ± SEM and were analyzed by the Shapiro–Wilk test to certify normal distribution. *F*-tests were used to determine variance. For simple comparisons, two-tailed Student’s *t*-test was used, whereas for multiple comparisons, ANOVA followed by the Tukey post-hoc test was utilized. Differences with *p* < 0.05 were considered significant.

## 3. Results

### 3.1. Sympathetic Overdrive in the rd10 Retina: Desensitization Defects

**Figure 1:**
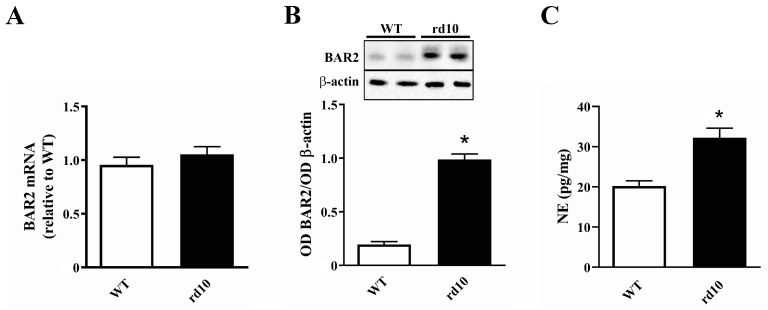
Expression of beta 2 adrenergic receptors (BAR2) and norepinephrine (NE) in the retina of wild type (WT) and rd10 mice. (**A**) Transcript levels of BAR2. (**B**) Protein levels of BAR2. (**C**) Levels of NE. BAR2 transcripts did not differ between WT and rd10 mice while BAR2 protein and NE were both upregulated in rd10 mice (*n* = 5 samples per group, each containing 2 retinas from independent mice). * *p* < 0.001 versus WT (two-tailed Student’s *t*-test).

In rd10 mice, BAR2 transcripts did not differ from those measured in WT mice (Figure 1A). In contrast, protein levels of BAR2 were significantly increased, with levels in rd10 mice that were 5.0-fold higher than those measured in WT (Figure 1B). Together, NE levels were 1.6-fold higher than those in WT (Figure 1C).

**Figure 2:**
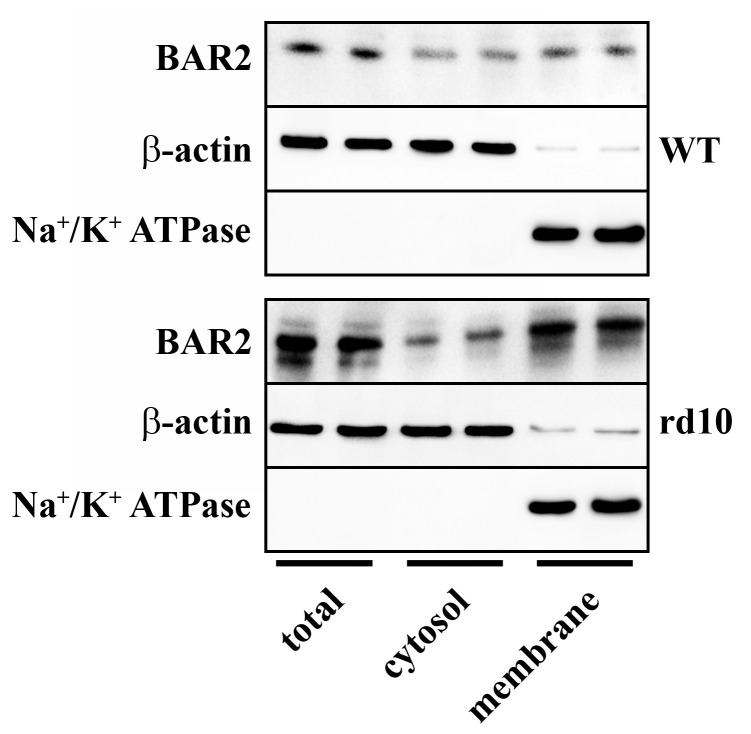
Expression of BAR2 in total extracts, cytosol fractions and plasma membrane fractions obtained from retinas of WT and rd10 mice. Representative Western blots from retinal homogenates are shown. In rd10 mice, BAR2 was upregulated in the plasma membrane fraction (*n* = 5 samples per group, each containing 2 retinas from independent mice).

Results shown in Figure 2 demonstrate that, in respect to WT mice, in rd10 mice BAR2 upregulation was mainly due to an increased plasma membrane fraction instead of cytosolic fraction.

**Figure 3:**
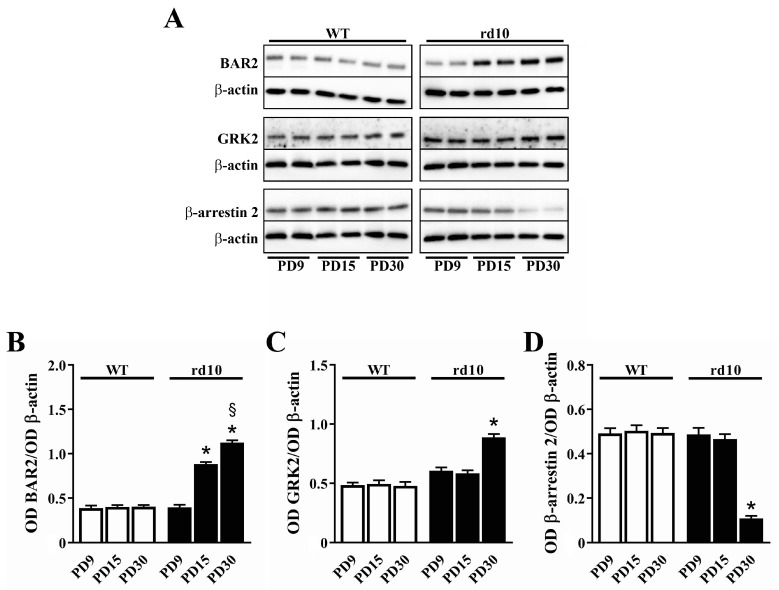
Developmental expression of BAR2, G protein-coupled receptor kinase (GRK)2 and β-arrestin 2 in the retina of WT and rd10 mice. (**A**) Representative Western blots from retinal homogenates. (**B**–**D**) Densitometric analysis of BAR2 (**B**), GRK2 (**C**) and β-arrestin 2 (**D**) levels. In WT mice, no differences in the levels of BAR2, β-arrestin 2 or GRK2 were observed from PD9 to PD30. In rd10 mice, BAR2 progressively increased. At PD30, GRK2 levels were drastically higher than at PD9, while β-arrestin 2 levels were drastically lower (*n* = 5 samples per group, each containing 2 retinas from independent mice). * *p* < 0.001 versus rd10 at PD9; ^§^ *p* < 0.001 versus rd10 at PD15 (one-way ANOVA followed by the Tukey post-hoc test).

The increase in BAR2 levels in rd10 mice is an event that parallels photoreceptor degeneration. As shown in Figure 3, in rd10 mice, BAR2 levels started to increase at PD15 in concomitance with the initial phase of rod death, while in WT mice, BAR2 levels remained constant from PD9 to PD30. At PD15 and PD30, BAR2 levels were 2.2- and 2.8-fold higher than at PD9, respectively. BAR2 upregulation was coupled to a slight increase in GRK2 (1.5-fold in respect to PD9) and a drastic decrease in β-arrestin 2 (4.5-fold in respect to PD9).

**Figure 4:**
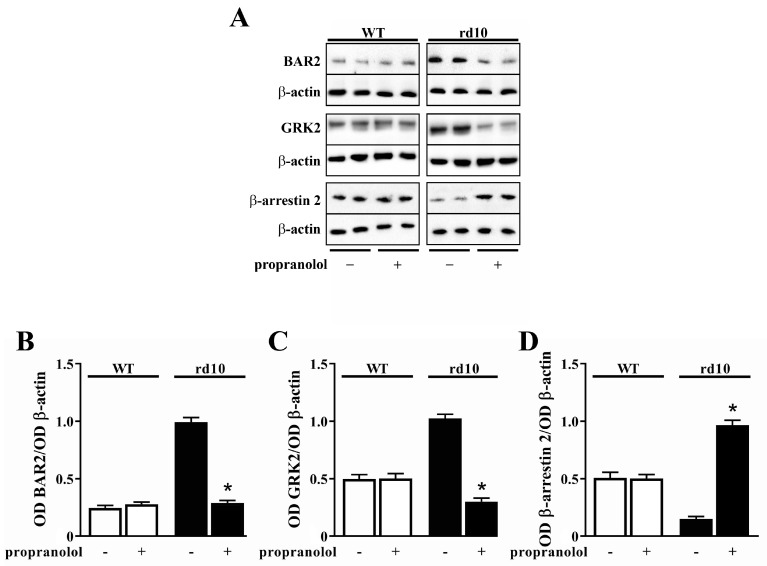
Expression of BAR2, GRK2 and β-arrestin 2 in the retina of WT and rd10 mice untreated or treated with propranolol. (**A**) Representative Western blots from retinal homogenates. (**B**–**D**) Densitometric analysis of BAR2 (**B**), GRK2 (**C**) and β-arrestin 2 (**D**) levels. In WT mice, propranolol did not affect the expression of BAR2, GRK2 or β-arrestin 2. In rd10 mice, propranolol reduced BAR2 and GRK2 levels, while increased β-arrestin 2 levels (*n* = 5 samples per group, each containing 2 retinas from independent mice). * *p* < 0.001 versus untreated rd10 (one-way ANOVA followed by the Tukey post-hoc test).

At PD30, when in rd10 mice most rods have degenerated and only some cones are still there [26,27], propranolol administration was effective in reducing BAR2 and GRK2 levels (by 3.4- and 3.3-fold, respectively), while it increased β-arrestin 2 levels (by 6.4-fold). WT levels of BAR2, GRK2 and β-arrestin 2 were not affected by propranolol (Figure 4). No statistical difference in the levels of BAR2 (*p* = 0.796; one-way ANOVA followed by the Tukey post-hoc test), GRK2 (*p* = 0.865; one-way ANOVA followed by the Tukey post-hoc test) and β-arrestin 2 (*p* = 0.827; one-way ANOVA followed by the Tukey post-hoc test) was observed between untreated and vehicle-treated mice (data not shown).

### 3.2. HIF-1 Regulation of BAR2 Expression

**Figure 5:**
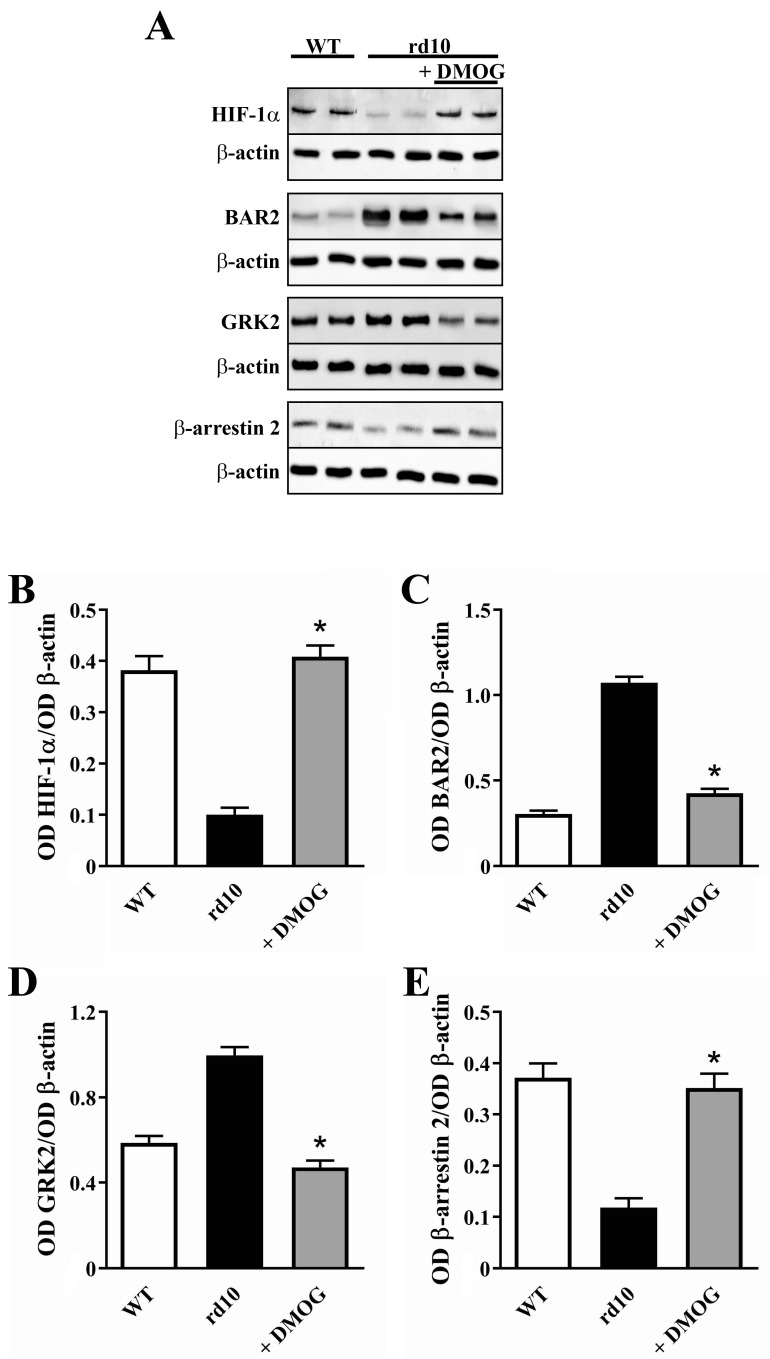
Expression of hypoxia-inducible factor (HIF)-1α, BAR2, GRK2 and β-arrestin 2 in the retina of untreated WT and rd10 mice either untreated or treated with dimethyloxalylglycine (DMOG). (**A**) Representative Western blots from retinal homogenates. (**B**–**E**) Densitometric analysis of HIF-1α (**B**), BAR2 (**C**), GRK2 (**D**) and β-arrestin 2 (**E**) levels. In rd10 mice, DMOG increased HIF-1α and β-arrestin 2 levels, while it reduced BAR2 and GRK2 levels (*n* = 5 samples per group, each containing 2 retinas from independent mice). * *p* < 0.001 versus untreated rd10 (one-way ANOVA followed by the Tukey post-hoc test).

The rd10 mouse model is characterized by a hyperoxic environment due to the low oxygen consumption by the outer retina in which photoreceptors are mostly degenerated [28,29]. High oxygen tension almost halves the levels of the oxygen sensitive transcription factor HIF-1 [5,6] that we hypothesized to influence BAR2 levels in the retina. We therefore explored the possibility that recovering HIF-1 levels by preventing HIF-1α degradation might hamper BAR2 upregulation. To this aim, HIF-1α degradation was inhibited by the administration of DMOG. As shown in Figure 5, DMOG increased the level of HIF-1α by 4.1-fold. In addition, DMOG downregulated BAR2 and GRK2 levels by 2.5- and 2.1-fold, respectively, while it upregulated β-arrestin 2 levels by 3.0-fold. No statistical difference in the levels of HIF-1α (*p* = 0.892; one-way ANOVA followed by the Tukey post-hoc test), BAR2 (*p* = 0.917; one-way ANOVA followed by the Tukey post-hoc test), GRK2 (*p* = 0.884; one-way ANOVA followed by the Tukey post-hoc test) and β-arrestin 2 (*p* = 0.905; one-way ANOVA followed by the Tukey post-hoc test) was observed between untreated and vehicle-treated mice (data not shown).

**Figure 6:**
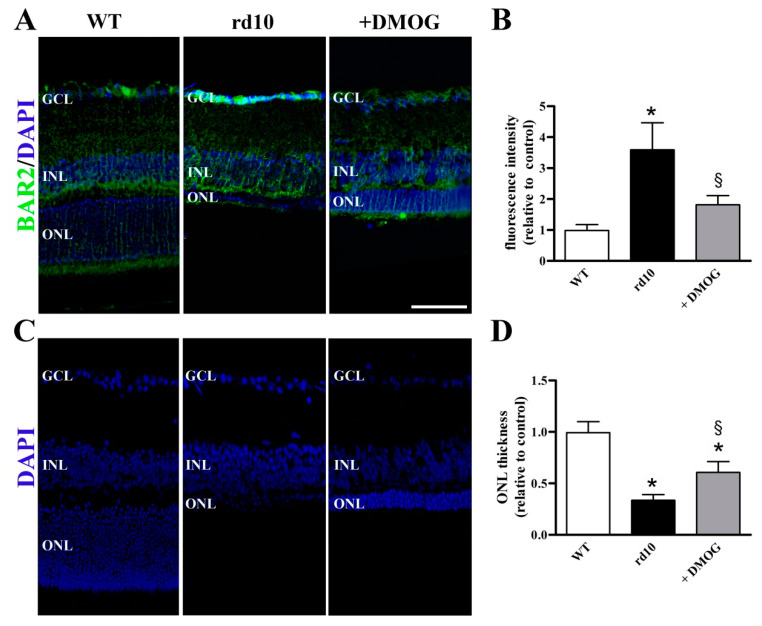
BAR2 localization in retinal sections from untreated WT and rd10 mice either untreated or treated with DMOG. (**A**) Images of retinal sections immunolabeled with antibodies against BAR2 (green) and stained with 4′,6-diamidino-2-phenylindole (DAPI; blue). (**B**) Quantitative analysis of fluorescence intensity. (**C**) Images of retinal sections stained with DAPI. (**D**) Quantitative analysis of the ONL thickness. DMOG decreased BAR2 immunoreactivity and increased the ONL thickness in rd10 mice. GCL, ganglion cell layer; INL, inner nuclear layer, ONL, outer nuclear layer. Scale bar, 50 µm (*n* = 5 retinas per group). * *p* < 0.001 versus WT; ^§^ *p* < 0.01 versus untreated rd10 (one-way ANOVA followed by the Tukey post-hoc test).

Decreased levels of BAR2 after DMOG administration were confirmed by immunolabeling experiments. In particular, in WT retinas, we observed BAR2 immunoreactive fibers passing through the ONL and some BAR2 immunoreactive processes in the ganglion cell layer (GCL). As compared to WT retinas, the rd10 retina showed more intense BAR2 immunolabeling at the level of both ONL and GCL. DMOG administration reduced BAR2 immunolabeling that became less intense in comparison with rd10 mice without DMOG (Figure 6A). Quantitative analysis of fluorescence intensity showed that BAR2 immunoreactivity was increased in rd10 mice by 3.6-fold, while it was reduced by DMOG by 2.0-fold (Figure 6B). As also shown in Figure 6C, the ONL demonstrated significantly less cells in rd10 retinas compared to WT retinas. DMOG administration significantly increased the ONL thickness by 2.2-fold (Figure 6D). No statistical difference in the fluorescence intensity (*p* = 0.776; one-way ANOVA followed by the Tukey post-hoc test) and in the ONL thickness (*p* = 0.844; one-way ANOVA followed by the Tukey post-hoc test) was observed between untreated and vehicle-treated mice (data not shown).

### 3.3. HIF-1α Stabilization Partially Prevents Reactive Gliosis, Microglial Activation and Cone Loss

**Figure 7:**
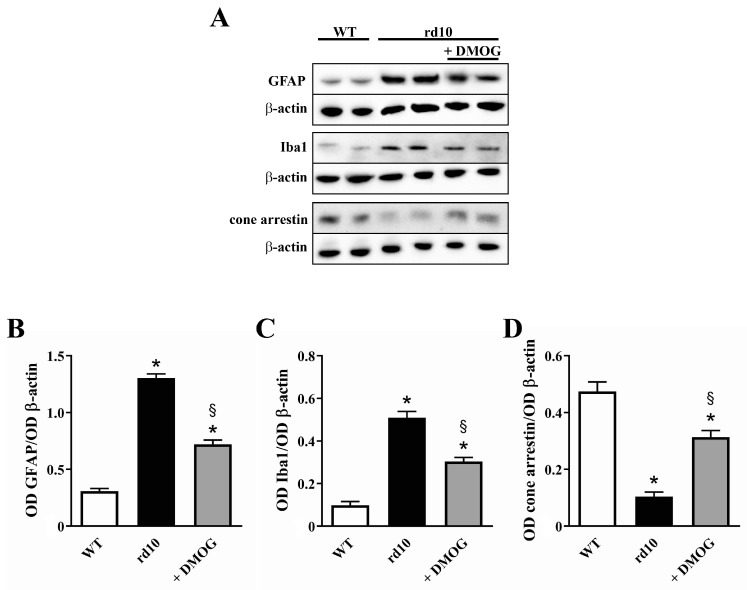
Expression of glial fibrillary acidic protein (GFAP), ionized calcium binding adaptor molecule 1 (Iba1) and cone arrestin in the retina of untreated WT and rd10 mice either untreated or treated with DMOG. (**A**) Representative Western blots from retinal homogenates. (**B**–**D**) Densitometric analysis of GFAP (**B**), Iba1 (**C**) and cone arrestin levels. In rd10 mice, DMOG decreased GFAP and Iba1 levels, while increased cone arrestin levels (*n* = 5 samples per group, each containing 2 retinas from independent mice). * *p* < 0.001 versus WT; ^§^ *p* < 0.001 versus untreated rd10 (one-way ANOVA followed by the Tukey post-hoc test).

Reactive gliosis and microglial activation are typical of retinas of rd10 mice [30] and additional animal models of RP [31,32]. As shown in Figure 7, the reactive gliosis and microglial activation were characterized by up-regulation of both GFAP and Iba-1, very sensitive early indicators of retinal stress in Müller cells [33] and activated microglial cells [34], by 4.2- and 5.2-fold, respectively. As expected, very low levels of cone arrestin were determined in rd10 mice (4.6-fold lower than in WT). As also shown in Figure 7, DMOG administration reduced protein levels of both GFAP and Iba1 (by 1.8- and 1.7-fold, respectively), while partially prevented the reduction in cone arrestin levels, which were 3.0-fold higher than in untreated rd10 mice. No statistical difference in the levels of GFAP (*p* = 0.922; one-way ANOVA followed by the Tukey post-hoc test), Iba1 (*p* = 0.877; one-way ANOVA followed by the Tukey post-hoc test) and cone arrestin (*p* = 0.784; one-way ANOVA followed by the Tukey post-hoc test) was observed between untreated and vehicle-treated mice (data not shown).

**Figure 8:**
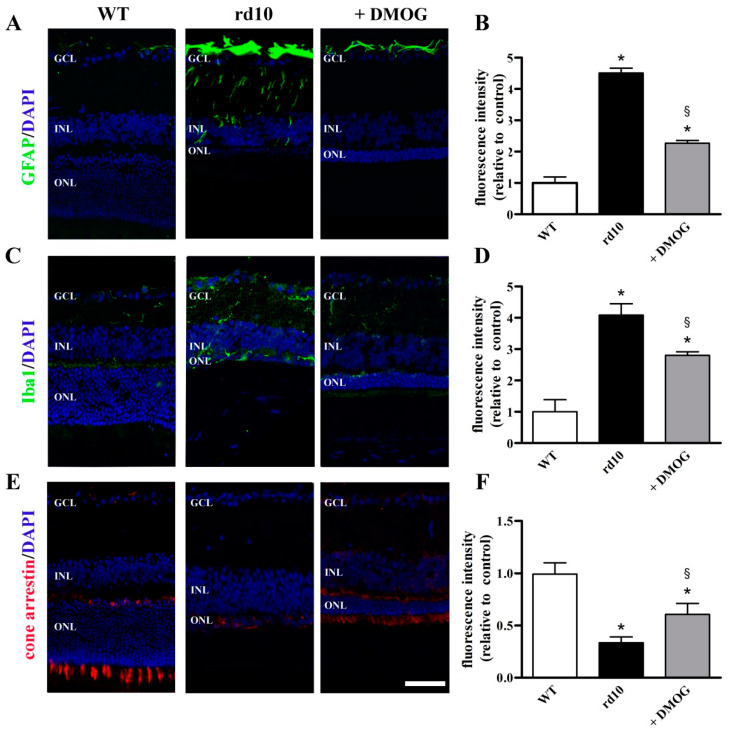
Localization of GFAP, Iba1 and cone arrestin in retinal sections from untreated WT and rd10 mice either untreated or treated with DMOG. (**A**) Images of retinal sections immunolabeled with antibodies against GFAP (green) and stained with DAPI (blue). (**B**) Quantitative analysis of fluorescence intensity. (**C**) Images of retinal sections immunolabeled with antibodies against Iba1 (green) and stained with DAPI (blue). (**D**) Quantitative analysis of fluorescence intensity. (**E**) Images of retinal sections immunolabeled with antibodies against cone arrestin (red) and stained with DAPI (blue). (**F**) Quantitative analysis of fluorescence intensity. In rd10 mice, DMOG decreased both GFAP and Iba1 levels, while increased cone arrestin levels. GCL, ganglion cell layer; INL, inner nuclear layer; ONL, outer nuclear layer. Scale bar, 50 µm (*n* = 5 retinas per group). * *p* < 0.001 versus WT; ^§^ *p* < 0.01 versus untreated rd10 (one-way ANOVA followed by the Tukey post-hoc test).

As shown in Figure 8, results from Western blot analysis were confirmed by immunohistochemistry. In fact, in WT retinas, GFAP labeling was restricted mainly to astrocytes located in the GCL layer while in the retina of rd10 mice Müller cells showed extensive GFAP immunolabeling along their processes which crossed the entire retina. DMOG administration reduced GFAP immunolabeling (Figure 8A). Quantitative analysis of fluorescence intensity showed that GFAP was increased in rd10 mice by 4.5-fold and that DMOG reduced the intensity by 2.0-fold (Figure 8B). In WT mice, Iba1 immunolabeling was localized to the inner retina, including the inner nuclear layer and the GCL. No Iba1 immunolabeling was observed in the ONL. In rd10 mice, Iba1 immunolabeling was found in all retinal layers particularly in the outer retina. DMOG administration reduced microglial activation as determined by decreased Iba1 immunoreactivity (Figure 8C). As shown by the quantitative analysis of fluorescence intensity, in rd10 mice, Iba1 was increased by 4.1-fold, while DMOG reduced Iba1 intensity by 1.5-fold (Figure 8D). As the last link in the chain, immunostaining for cone arrestin, an essential protein in the cone visual transduction cascade, was extremely low in rd10 retinas, while it revealed substantial preservation of cone cells in rd10 mice treated with DMOG as compared to untreated mice (Figure 8E). As shown by the quantitative analysis of fluorescence intensity, in rd10 mice, cone arrestin was reduced 3.0-fold, while DMOG increased cone arrestin intensity by 1.8-fold (Figure 8F). No statistical difference in the fluorescence intensity of GFAP (*p* = 0.794; one-way ANOVA followed by the Tukey post-hoc test), Iba1 (*p* = 0.944; one-way ANOVA followed by the Tukey post-hoc test) and cone arrestin (*p* = 0.877; one-way ANOVA followed by the Tukey post-hoc test) was observed between untreated and vehicle-treated mice (data not shown).

### 3.4. Effects of HIF-1α Stabilization on ERG

**Figure 9:**
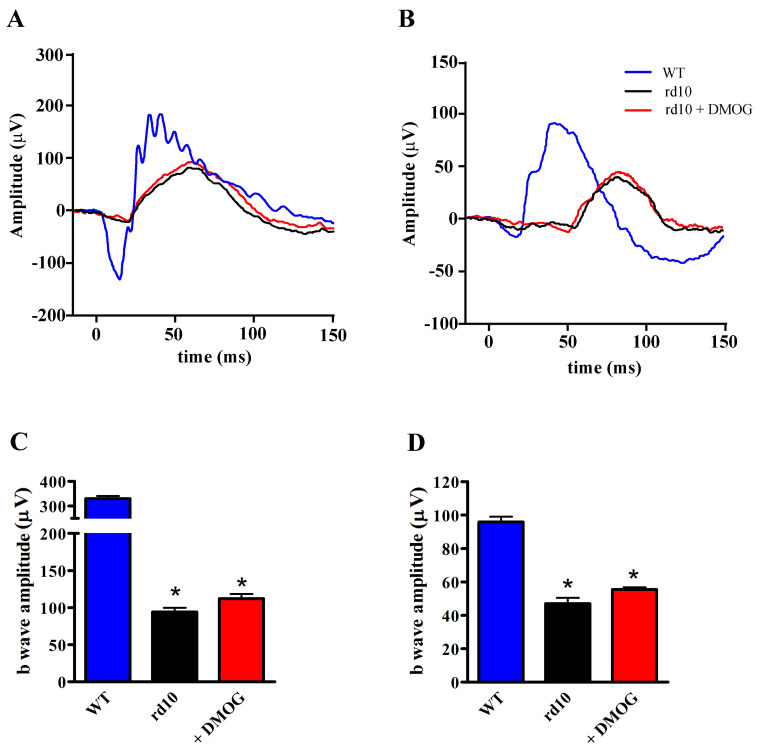
Electroretinogram (ERG) responses in untreated WT and rd10 mice either untreated or treated with DMOG. (**A**,**B**) Representative ERG traces recorded at PD30 at light intensity of 1 log cd-s/m^2^ in scotopic (**A**) or photopic (**B**) conditions. (**C**,**D**) Average peak amplitudes of scotopic rod/cone (**C**) and photopic (**D**) b-waves. DMOG did not restore b-wave amplitudes (*n* = 5 mice per group). * *p* < 0.001 versus WT (one-way ANOVA followed by the Tukey post-hoc test).

To evaluate whether protective effects of DMOG were paralleled by improved visual function, DMOG-treated rd10 mice were subjected to ERG analysis to assess rod and cone function. As shown by ERG responses in Figure 9A (scotopic rod/cone responses) and 9B (photopic responses), small and variable a-waves could be detected in rd10 mice without any difference between untreated and DMOG-treated animals. As a-waves could not be measured reliably, we chose b-waves as an indirect measure of photoreceptor function. Quantitative analysis of the b-wave amplitude is shown in Figure 9C (scotopic rod/cone responses) and 9D (photopic responses). Responses from rd10 mice were compared with WT mice. WT mice had robust photoreceptor responses, whereas in rd10 mice weak photoreceptor responses could be detected. At 1.00 log cd-s/m^2^, in rd10 mice treated with DMOG, the amplitude of the scotopic and photopic b-waves was about 34% and 58% of the corresponding amplitude in WT mice, respectively, without any significant difference with untreated animals. No statistical difference in the b wave amplitude both in scotopic (*p* = 0.952; one-way ANOVA followed by the Tukey post-hoc test) and photopic (*p* = 0.894; one-way ANOVA followed by the Tukey post-hoc test) conditions was observed between untreated and vehicle-treated mice (data not shown).

## 4. Discussion

In the rd10 model of RP, the BAR antagonist metipranolol efficiently counteracts retinal damage by reducing nitrosative stress, which contributes to cone cell death [11]. In order to increase the understanding of the mechanisms underlying the efficacy of BAR blockade, here we demonstrate that the sympathetic system is overstimulated in the rd10 retina. Mechanisms underlying BAR2 overexpression were also determined and the efficacy of the BAR blocker propranolol was assessed. In addition, a possible involvement of HIF-1 in regulating BAR2 expression was determined. Some evidence was also provided to support the hypothesis that, in RP, sympathetic overstimulation might be coupled to neuroinflammatory processes leading to cone photoreceptor loss.

### 4.1. Overactivation of the Sympathetic System

As shown by the present results, the retina of rd10 mice is characterized by overactivation of the sympathetic system, a finding that is in line with the efficacy of BAR1/2 blockers against rd10-associated retinal damages [11]. In particular, BAR2 proteins are drastically upregulated while BAR2 mRNA remains unaltered, indicating that overexpressed BAR2 proteins are not dependent on altered BAR2 transcription. Rather, our data suggest that BAR2 upregulation is likely to depend on defects of the desensitization process due to β-arrestin 2 downregulation, which uncouples the receptor from G proteins thus resulting in BAR2 accumulation at the cell membrane. Altered ligand-induced BAR2 desensitization, as for instance after inhibiting GRK2, blocks agonist-induced desensitization in pathogenic and clinical settings [35]. Previous studies have demonstrated that BAR2 is expressed by Müller cells in the mouse retina and by cultured Müller cells of the rat retina [18,19]. The present findings that in rd10 mice BAR2 immunolabeling is increased in INL (in which Müller cell bodies are localized), ONL (where Müller cells processes surround the photoreceptor cell bodies) and GCL (in which Müller cell endfeet are localized) support the possibility that upregulated BAR2 is indeed localized to Müller cells, although additional cells not yet identified may also express BAR2 at the plasma membrane.

As shown by the present results, rd10 mice are also characterized by NE accumulation that can be interpreted as an augmented transmitter release from sympathetic nerves directed to choroidal vessels from which NE enters the retina and/or from retinal cells that produce catecholamines. In fact, pathologic conditions may represent a potent stimulus for NE production not only via sympathetic nerves, but also via intrinsic production by retinal neurons and/or endothelial cells [17].

The functional significance of sympathetic overstimulation raises some critical questions. For instance, is the sympathetic system overstimulated as a consequence of pathologic conditions that trigger major inflammatory processes? A positive link between sympathetic overstimulation and inflammation is well established [36] although overstimulation of the sympathetic system may be also interpreted as a compensatory mechanism to dampen the neuroinflammatory response [37]. Increased sympathetic transmission may also be coupled to vasomotor responses that regulate the blood flow in the diseased retina. The rd10 model, for instance, is characterized by reduced blood flow, which is secondary to a decreased demand for supply [38]. In this respect, overstimulation of the sympathetic system may serve as a compensatory mechanism to counteract the reduced blood flow. In human umbilical veins, high oxygen levels appear to be responsible for endogenous NE release, which may account for the vascular tone that contributes to the regulation of blood flow from the placenta to the fetus [39].

In the rd10 model, the BAR1/2 blocker metipranolol prevents the nitrosative damage induced by the reaction between nitric oxide and oxygen free radicals, which leads to cone death [11]. Metipranolol is also known to decrease retinal cell death through reduced nitrosative stress in additional models of retinal degeneration [40]. However, the possibility that the efficacy of BAR blockade may depend on BAR blocker interaction with the BAR system has not been evaluated yet. Here, we used an additional BAR1/2 blocker, propranolol, which, at variance with metipranolol, appears to be devoid of any free radical scavenger activity [41]. As shown by the present study, upregulated levels of BAR2 in RP are drastically reduced by propranolol, suggesting that BAR blockade triggers a negative feedback loop that downregulates the levels of its target, which probably would make the treatment more effective than if acting only at the signal transduction level. Reduced levels of BAR2 are likely to depend on restored desensitization processes presumable due to an increased expression of β-arrestin 2, which drives BAR2 internalization. In this respect, in bronchial epithelial cells challenged with cigarette smoke, propranolol reverses the smoke-induced downregulation of β-arrestin 2, while in kidney cells overexpressing BAR2, propranolol stimulates the recruitment of β-arrestin 2 to the receptor [42,43]. According to the established propranolol efficacy in reducing BAR2 levels, the possibility that the drug may act in preventing cone loss can be assumed although the molecular mechanisms involved in the protective effect of propranolol need to be fully elucidated.

### 4.2. HIF-1α Stabilization Reduces BAR2 Upregulation and Restores the Desensitization Cascade

In RP, photoreceptor degeneration leads to a rise in oxygen levels, which creates a hyperoxic retinal microenvironment [2]. In a rat model of RP, for instance, oxygen levels are increased by about 50% in the outer retinal layers [3] thus causing downregulation of the HIF-1/VEGF axis [5,6], which, in turn, leads to retinal vessel attenuation [6,29]. In the rd10 model of RP, stabilization of HIF-1α via prolyl hydroxylase inhibition with the 2-oxoglutarate analog DMOG recovers at least in part the retinal levels of HIF-1/VEGF [5]. As shown here, preventing the degradation of HIF-1α through DMOG administration reduces BAR2 upregulation, which is indicative of the possibility that HIF-1 may regulate BAR2 expression. However, the lack of BAR2 messenger regulation seems to exclude that HIF-1 is coupled to BAR2 transcription. Rather, we demonstrate that preventing HIF-1α drop restores the correct desensitization cascade of BAR2, indicating a link between HIF-1 and BAR2 upregulation. In this respect, the growth of cancer cells under chronic stress appears to be regulated by the HIF-1/BAR2 axis as demonstrated by the finding that resveratrol inhibits the proliferation of chronically stressed cancer cells by preventing the upregulation of both HIF-1α and BAR2 [44]. Another possibility is that DMOG acts directly on BAR2 internalization without the intervention of HIF-1. As described above, DMOG is an inhibitor of prolyl hydroxylases, the enzymes responsible for the hydroxylation of HIF-1α, which allow the protein to be targeted for degradation [7] and there is evidence that prolyl hydroxylases can directly modulate the rate of BAR2 internalization through interactions with β-arrestin 2 [45]

### 4.3. HIF-1α Stabilization Restores the Neuroinflammatory Cascade and Prevents Cone Loss

Stabilization of HIF-1α decreases GFAP, an indicator of Müller cells gliosis whose upregulation in response to retinal stress is likely to be a cellular strategy to protect the retina and promote its repair [31]. Likewise, stabilization of HIF-1α reduces upregulated levels of Iba1, indicating decreased microglial activation in response to retinal stress. These results are in line with the finding that inappropriate levels of HIF-1α are coupled to a cascade of events leading to cone photoreceptor damage [6]. The additional fact that cone rescue is not sufficient to recover the amplitude of the ERG photopic response remains to be clarified, although the anatomical rescue of the retina is not always coupled to its functional recovery [46,47]. For instance, in a mouse model of RP, partial preservation of photoreceptor structure does not rescue completely the retinal function [48]. On the other hand, persisting conditions including abnormal synaptic connections after rod degeneration and/or the loss of rod-derived factors that support cone survival may prevent the rescue of retinal function [49].

## 5. Conclusions

**Figure 10:**
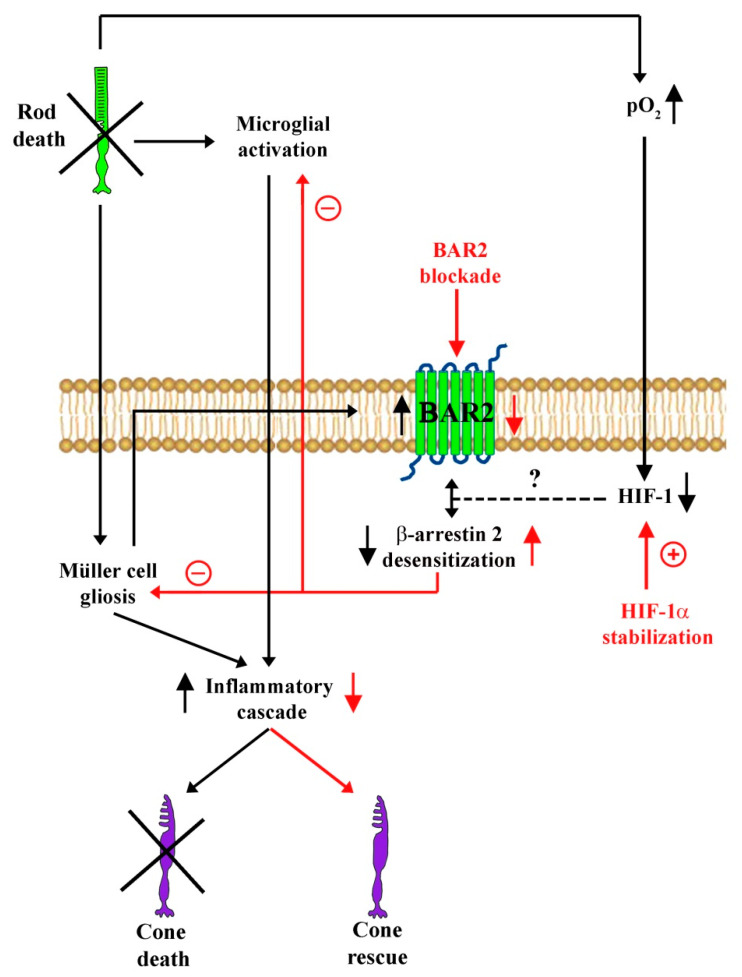
Schematic diagram depicting hypothetical mechanisms through which preventing BAR2 upregulation counteracts cone cell death in a mouse model of retinitis pigmentosa. Genetically determined rod death triggers microglial activation and Müller cell gliosis resulting in BAR2 overexpression in Müller cells likely through the inhibition of desensitization processes. At the same time, the hyperoxic environment produced by photoreceptor death leads to HIF-1α degradation that contributes to the lack of desensitization. The noxious environment induces cone death, which progressively worsens retinal function. Either BAR2 blockade with propranolol or HIF-1α stabilization with DMOG recover desensitization processes thus counteracting cone cell death. Black arrows indicate the events consequent to rod degeneration, while red arrows indicate the events consequent to BAR2 blockade and/or HIF-1α stabilization. The dotted line indicates the hypothetical involvement of HIF-1 in BAR2 upregulation.

In summary, the present data support the possibility that preventing BAR2 upregulation through BAR2 blockade or HIF-1α stabilization reduces cone photoreceptor death in rd10 mice although the HIF-1α results are correlative, but not proof of causation. The schematic diagram of Figure 10 summarizes the cascade of events triggered by rod degeneration and hyperoxic retinal environment. The finding that both propranolol and DMOG recover desensitization processes allows to speculate that upregulated levels of BAR2 are somehow coupled to cone photoreceptor loss, although the underlying mechanisms remain to be clarified. Our hypothesis is that preventing sympathetic overstimulation may be beneficial for patients with retinal degenerations, either alone or in combination with other therapies. Although the effect of DMOG on BAR2 upregulation is interesting, further mechanistic studies are needed to clarify the hypothesis that sympathetic overactivation is somehow coupled to cone photoreceptor death. In particular, a whole RNA sequencing approach, which, for instance, has been successfully used to explore the role of oxidative stress in RP etiopathogenesis [8,50,51], may be used to analyze differences in the transcriptome of WT and rd10 retina and to deeply investigate the mechanisms linking BAR2 to cone death, especially focusing on regulatory processes involving HIF-1 and additional interactors.

## Supplementary Materials

The following are available online at https://www.mdpi.com/2073-4409/9/9/2060/s1, Table S1. Sequences of primer pairs used for qPCR experiments; Table S2: List of antibodies used in Western blot analysis.
