# Supplementary material for: Novel Insights into Beta 2 Adrenergic Receptor Function in the rd10 Model of Retinitis Pigmentosa
Source: Cells. 2020 Sep 9;9(9):2060. doi: 10.3390/cells9092060 (PMC7563182; doi:10.3390/cells9092060)
Supplement: Supplementary file 1 [file cells-09-02060-s001.pdf]

Table 1. Sequences of primer pairs used for qPCR experiments.

Gene	Primer sequence (5′ –> 3′)	
	**Forward Primer**	**Reverse Primer**
BAR2	GGGAACGACAGCGACTTCTT	GCCAGGACGATAACCGACAT
Rpl13a	CACTCTGGAGGAGAAACGGAAGG	GCAGGCATGAGGCAAACAGTC

Table S2. List of antibodies used in Western blot analysis.

Antibody	Dilution	Source	Cat. No.
Mouse monoclonal anti-BAR2	1:200	Santa Cruz Biotechnology	sc-81577
Rabbit polyclonal anti GRK2	1:1,000	Abcam	ab137666
Goat polyclonal anti β-arrestin 2	1:1,000	Abcam	ab31294
Rabbit polyclonal anti HIF1-α	1:500	Abcam	ab2185
Rabbit monoclonal anti-Iba1	1:1,000	Abcam	ab178846
Mouse monoclonal anti-GFAP	1:1,000	Sigma-Aldrich	G3893
Rabbit polyclonal anti-cone arrestin	1:500	Sigma-Aldrich	AB15282
Mouse monoclonal anti-β-actin	1:2,500	Sigma-Aldrich	A2228
Rabbit polyclonal anti-Na^+/K^+ ATPase	1:1,000	Cell Signaling Technology	#3010
